# Diagnosis and impact of neuropathic pain in leprosy patients in Nepal after completion of multidrug therapy

**DOI:** 10.1371/journal.pntd.0006610

**Published:** 2018-07-02

**Authors:** Han-Siong Toh, Jeni Maharjan, Ruby Thapa, Kapil Dev Neupane, Mahesh Shah, Suwash Baral, Deanna A. Hagge, Indra Bahadur Napit, Diana N. J. Lockwood

**Affiliations:** 1 Department of Intensive Care Medicine, Chi Mei Medical Center, Tainan, Taiwan; 2 Mycobacterial Research Laboratories, Anandaban Hospital, The Leprosy Mission Nepal, Kathmandu, Nepal; 3 Department of Clinical Research, London School of Hygiene and Tropical Medicine, London, England; University of California San Diego School of Medicine, UNITED STATES

## Abstract

**Objectives:**

Neuropathic pain (NP) can occur as a chronic complication of leprosy neuropathy. NP epidemiology and its impact on patients have not been well documented. This study investigates NP prevalence and impact in the years after patients are declared “released from treatment” (RFT) following multidrug therapy (MDT) completion.

**Methods:**

In this cross-sectional study, 85 RFT patients were recruited within leprosy referral services in Nepal. The Douleur Neuropathique 4 Questionnaire (DN4) was used to screen for NP. Pain severity, impacts on patients’ daily activities and mental health were measured by using the Brief Pain Inventory (BPI), Screening of Activity Limitation and Safety Awareness (SALSA), and General Health Questionnaire-12 (GHQ-12) respectively.

**Results:**

96% surveyed had been treated for multibacillary leprosy. 44 (52%) complained of pain of which 30 (68%) were diagnosed with NP. NP was not associated with age, gender, or presence of skin lesions or nerve symptoms at leprosy diagnosis. 70% of patients with NP had either history of or ongoing reactions and 47% had grade 2 disability. Nerve tenderness (*p* = 0.023) and current reactions (*p* = 0.018) were significant risk factors for NP. Patients with NP suffered significantly higher intensity pain (*p* = 0.023) and daily life interference (*p* = 0.003) and were more likely to have moderate to extreme daily activity limitations (*p* = 0.005). 13 (43%) exhibited psychological distress, and medications only reduced moderate degree (50–60%) of pain.

**Conclusions:**

In our study, 35% of RFT patients had ongoing NP. Risk factors include nerve tenderness and reaction. They suffer from more daily life interference and psychological distress. Leprosy patient care should include recognition and management of NP.

## Author summary

Leprosy could present with chronic neuropathic pain, which might severely disturb daily life and mental health of leprosy-infected patients. This complication could also occur even years after antibiotics therapy completion, and we noticed that it is associated with recent leprosy reactions and nerve tenderness on physical examination. This is the first study that describes the epidemiology of neuropathic pain in leprosy patients in Nepal. More than one-third of leprosy patients who had completed multidrug therapy, are still suffering from neuropathic pain, and this study demonstrated that it has caused a great impact to their daily activities and psychological status. Therefore, prompt recognition and management of neuropathic pain are mandatory while taking care of the patients with leprosy.

## Introduction

Leprosy, also known as Hansen's disease, is a chronic infectious disease caused by *Mycobacterium leprae* (*M*. *leprae*). It is characterized by the inflammation of skin and nerve, which could lead to disabling peripheral neuropathy. Since the introduction of multidrug therapy (MDT), leprosy became a treatable disease; and its prevalence decreased globally over the past two decades. However, it continues to persist as an important public health issue in endemic areas [1]. As one of the 13 most highly endemic countries, more than 3000 new cases are diagnosed annually in Nepal; and 18 districts still report prevalence rates higher than 1/10,000 population in 2016 [2].

Nerve involvement in leprosy, or leprosy neuropathy, is primarily caused by the infection of Schwann cells by *M*. *leprae* which can leave persistent antigen capable of stimulating inflammatory episodes years after cure. Inflammatory or immune complication episodes are called leprosy reactions and are classified into three types: neuritis (nerve involvement only), Type 1 reactions (T1R, Th1-mediated inflammation of skin with possible nerve involvement) and Erythema Nodosum Leprosum (ENL, occurs in high *M*. *leprae* burden patients, involving skin with possible nerve involvement). Leprosy reactions can affect up to 30–50% of leprosy patients and can, therefore, initiate or progress leprosy neuropathy at any time before diagnosis, during treatment, and even years after MDT [3, 4].

Clinically, leprosy neuropathy can present with nerve thickening, sensory, autonomic or motor impairment and pain. The most frequently affected peripheral nerve is the ulnar nerve, and multiple nerve involvement is common especially in patients with high *M*. *leprae* burden [5]. Neuropathic pain (NP), which is defined as “pain caused by disease or disorders of the somatosensory nervous system”, can present concomitant with leprosy reaction or as a chronic and late-onset pain in leprosy patients [6].

As a non-visible and possibly late complication, NP can be difficult to diagnose; and its mechanisms are different from nociceptive pain. Continuous intraneural inflammation is believed to be the cause of small-fiber nerve damage, which could result in central and peripheral sensitization leading to chronic pain [7, 8]. Meanwhile, nerve thickening and tenderness have been documented to be related to NP, suggesting that ongoing inflammation in peripheral nerves might be responsible for the pain [9]. NP is usually moderate to severe, and it is commonly found in patients who had completed MDT [10, 11].

Studies in Brazil have demonstrated a prevalence of about 56% to 64% for NP in all leprosy patients, clinically defined as characteristic pain with associated symptoms along a leprosy-affected nerve distribution [12–14]. However, there is no consensus yet for making NP diagnosis in leprosy patients [15]. The guidelines of the International Association for the Study of Pain (IASP) recommend NP screening by Douleur Neuropathique 4 Questionnaire (DN4), which is one of the most commonly used screening tools in epidemiological studies of leprosy neuropathy [7, 13, 15]. In a study in Mumbai, 21% of “released from treatment” (RFT) patients were found to have NP [9]. Similar studies had been performed in Brazil, Ethiopia, China and Indonesia with findings that more than 20% of patients were reported to have NP [9–14, 16–23]. Due to the differences in screening tools, types of patients studied and study setting, the prevalence of NP in RFT patients varied from 11.3% to 70.3% [19, 21].

The presence of NP can severely affect a patient’s quality of life and generate psychological distress [12, 18]. Approximately 41% of leprosy patients with NP in the Mumbai study were found to have psychological distress, while a higher percentage of 76% was noted in Brazil [9, 18]. Another Brazilian study using the World Health Organization (WHO) Quality of Life Assessment BREF (WHOQoL-BREF) revealed that leprosy patients with NP also had lower quality of life scores in physical (*p* < 0.001) and psychological health (*p* = 0.04) domains compared to patients with nociceptive pain [12].

In terms of activity limitation, Arco et al. found about 90% of leprosy patients with NP had Grade 1 or 2 disability, and there is an association between NP and degree of disability (*p* < 0.05) [23]. Although the Screening of Activity Limitation and Safety Awareness (SALSA) scale has been recommended to measure the degree of limitation imposed by leprosy in daily life activities [24], only one study in Indonesia applied SALSA in correlation with NP [20]. The study demonstrated that two-thirds of leprosy patients with NP (12 in 18) had moderate to severe daily activities restrictions.

Thus, NP is an important complication of leprosy which is usually associated with negative impact on patients’ daily life, physical and psychological well-being. The prevalence of NP in people affected with leprosy has not been studied in many endemic countries, including Nepal. We hypothesized that NP remains a severe problem in Nepalese RFT patients. In order to evaluate this population, RFT patients associated with a referral hospital were interviewed and screened for NP using the DN4 then evaluated with the short form of Brief Pain Inventory (BPI-short) for pain severity; Eye, Hand and Foot (EHF) assessment and WHO grading for disability severity; SALSA for impact on activities; the General Health Questionnaire-12 (GHQ-12) for psychiatric morbidity alongside collection of leprosy demographics obtained from medical chart review.

## Materials and methods

### Study design

This cross-sectional observational study was conducted using screening tools and questionnaires to obtain quantitative data from RFT patients who had completed MDT at Anandaban Hospital in Kathmandu, Nepal. The hospital is the central referral hospital for leprosy complications management in Nepal with a patient base reporting from across the nation. Purposive sampling of RFT patients was performed in one month period (August of 2016) based on medical records from the inpatient ward, outpatient clinic and, the monthly satellite leprosy clinics in the southern flatlands (Terai) region.

### Ethics statement

The following criteria were employed for the recruitment: leprosy patients 18 years old or above, who completed MDT (RFT) and agreed to sign voluntary informed consent to participate in the study. This study had been approved by the Ethics Committee of the London School of Hygiene and Tropical Medicine (LSHTM; MSc Ethics Ref: 11141) and the Nepal Health Research Council (NHRC; Reg. no.161/2016).

### Data collection

**Fig 1:**
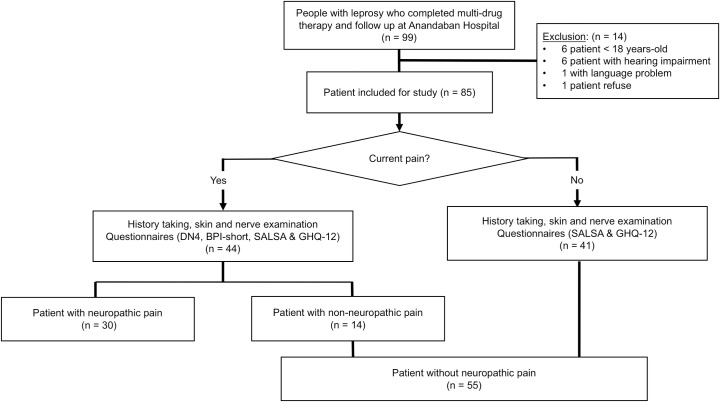
Flow-chart of patient enrolment and data collection.

All patients were interviewed in Nepali or Hindi language according to patient preference. Demographic data and a detailed history of leprosy were collected from the medical chart and interview. Comprehensive leprosy diagnosis according to WHO definitions had been based upon clinical exam with lesion count, physiotherapist exam for neuropathy, slit skin smear reports for bacterial index and skin biopsy histopathology. Leprosy is diagnosed clinically in patients with one or more of the following signs: anaesthetic skin lesions, thickened peripheral nerves, or acid-fast positive in skin smear or biopsy. [5] The WHO definition of leprosy classification is based on the number of skin lesions and skin smear findings. Paucibacillary (PB) leprosy is diagnosed in patients with five or fewer skin lesions. Multibacillary (MB) leprosy is diagnosed in patients with six or more skin lesions or positive skin smear. The results of histopathologic finding and Ridley-Jopling classification were also collected for further analyses. Similarly, chart reviews were used to identify leprosy reactions which had been diagnosed clinically by a dermatologist, with a record of new skin lesions or nerve symptoms at the time of presentation along with respective treatment regimen and duration. T1R was diagnosed in patients with acute inflammation in existing skin lesions, the development of nerve tenderness or new nerve function impairment. T2R was diagnosed in patients who developed erythematous tender skin papular or nodular lesions associated with systemic symptoms including fever. Neuritis was diagnosed in patients with reaction who developed nerve tenderness or pain. EHF score and WHO disability grade were recorded according to the WHO operational definitions [25]. Both eyes, hands and feet were evaluated and graded for severity of disability, and the EHF score was calculated with the sum of the gradings. The flow-chart of patient enrolment and data collection is depicted in Fig 1.

### Evaluation of pain

All patients were asked about a history of leprosy-related pain. However, only the patients who reported current pain were assessed by the DN4 questionnaire for NP and required to complete BPI-short for the evaluation of pain severity and inventory of medications taken for pain control. BPI-short includes a body chart for the localization of the area with pain, four severity items with numerical pain rating scale, a measure of response to medications and an evaluation of pain interference with seven items. Regarding the DN4 criteria, light finger touch and Semmes-Weinstein monofilaments were applied to the area of pain to determine touch hypoesthesia and pinprick hypoesthesia respectively [26]. Cotton wool was used to check for the presence of allodynia due to brushing.

### Evaluation of life impact

All patients, with or without current pain, were asked to complete the SALSA and the GHQ-12 questionnaires for the assessment of daily activity limitation and psychiatric morbidity, respectively. Both questionnaires had Nepalese versions available that had previously been used in leprosy investigations [27–29]. The Likert-type method was used to calculate the GHQ-12 score with the mean score of the study population applied as the threshold for psychological morbidity [30].

### Definitions

In accordance with DN4, NP was defined as pain with a score of 4 or more based on the DN4 criteria (DN4 ≥ 4) [26]. There are ten items in the questionnaire, and seven of them are about pain characteristics and associated symptoms (burning, painful cold, electric shock, tingling, pins and needle, numbness, itching). The other three items are to demonstrate touch or pinprick hypoesthesia and allodynia with clinical examination in the area of pain. The diagnosis of NP was confirmed by pain characteristics (patient’s description and questionnaire) and the occurrence of pain in a somatosensory dysfunction area (demonstration of hypoesthesia and allodynia by physical examination). For comparison to NP patients, patients with pain that scored less than 4 on the DN4 were regarded as a non-neuropathic pain (non-NP) control group. Patients with nociceptive pain were not specified as it is sometimes difficult to differentiate patients with mixed pain. RFT patients are defined as those who are technically cured of leprosy by having completed MDT (which might have been either 6, 12 or 24 months duration depending on disease classification and clinical discretion). “Nerve impairment at diagnosis” is defined as the presence of neuritis or neuropathy before the diagnosis of leprosy. The symptoms might be numbness, muscle weakness or nerve pain. Ulcers or other complications due to anesthetic hands or feet were also included. Nerve function impairment (NFI) was defined as the occurrence of motor or sensory function loss in peripheral nerves. In this study, motor function loss was defined as the weakness in any tested muscle with muscle power lower than 5, and sensory function loss as the failure to sense the monofilament test at two or more testing point. [25] Pain severity and its daily life interference were categorized into mild (1–4), moderate (5–6) and severe (7–10) according to the numerical rating scale of BPI-short [31]. Concerning activity restrictions, SALSA score was calculated by summing the scores of the 20 questions. Participants with a total SALSA score ≤ 24 were classified as without limitation, with scores for the degrees of limitation as follows: mild 15–39, moderate 40–49, severe 50–59 and extreme ≥ 60 [28].

### Statistical analyses

Statistical analyses were performed by using commercial statistical software STATA version 12.1 (StataCorp LP, College Station, Texas, United States). Comparisons between NP and patients without NP or non-NP group were analyzed using the Chi-square test or Fisher’s exact test for categorical variables, and t-test or Mann-Whitney test for continuous variables. A *p*-value < 0.05 was considered as significant evidence. Variables with a *p*-value less than 0.05 were chosen for multivariate logistic regression, using the stepwise forward method.

## Results

### Enrolment and general demographics

99 RFT patients were initially screened from hospital wards or various clinics within Nepalese leprosy referral services in August of 2016. Six patients under 18 years old were excluded as were 6 patients with severe hearing impairment due to aging and 1 patient who understood neither Nepali nor Hindi. Only 1 eligible patient refused to participate.

Following informed consent, 85 RFT patients were examined and questioned. The mean age was 43 ± 14 years and 64% (54/85) were male. Most RFT patients (96%, 82/85) had MB; and Ridley-Jopling classifications were primarily 44% (37/85) borderline tuberculoid (BT), 21% (18/85) borderline lepromatous (BL) and 25% (21/85) lepromatous (LL). Most (67%, 57/85) were within five years of MDT completion. Just over half (54%, 46/85) had a history of reactions, and one-third (34%, 29/85) were in reaction at the time of interview: 17 with ENL, 3 with T1R, and 9 with neuritis. 66 (78%) had NFI and 47 (55%) had grade 2 disability (G2D).

### Prevalence of neuropathic pain

65 (76%) of RFT patients had experienced leprosy-related pain in the past. During the interview, 44 (52%) complained of current pain and 30 (35%) had NP according to DN4 criteria (Table 1). 14 of them with pain but did not match the criteria of NP were denoted as the non-NP group in the pain severity analysis. The mean age of RFT patients with NP was 45 ± 15, of which 16 (53%) were male. BT type leprosy comprised the majority group (12/30, 40%) while LL and BL types were 23% (7/30) and 27% (8/30) of the patients respectively. 70% (21/30) of patients with NP had either history of or ongoing reactions with half (15/30, 50%) actively receiving management for reactions including prednisolone.

**Table 1:** Demographic data of study participants.

Variables	Patients with neuropathic pain (n = 30)	Patients without neuropathic pain (n = 55)	*p* value
Age, mean ± SD	45 ± 15	41 ± 14	0.174
Sex, N (%)			0.082
Male	16 (53)	38 (69)	
Female	14 (47)	17 (31)	
Type of leprosy, N (%)			1.000^#^
Multibacillary	29 (97)	53 (96)	
Paucibacillary	1 (3)	2 (4)	
Ridley-Jopling classification, N (%)			0.841^#^
Pure neuritic leprosy	2 (7)	5 (9)	
Borderline tuberculoid	12 (40)	25 (45)	
Borderline lepromatous	7 (23)	11 (20)	
Lepromatous leprosy	8 (27)	13 (24)	
Others	1 (3)	1 (2)	
Time to diagnosis, N (%)			0.667
≤ 6 months	14 (47)	23 (42)	
> 6 months	16 (53)	32 (58)	
Symptoms at diagnosis, N (%)			
Skin lesions	24 (80)	37 (67)	0.213
Nerve Function Impairment	20 (67)	37 (67)	0.955
Age of diagnosis, mean ± SD	35 ± 13	32 ± 15	0.372
Year since diagnosis, median (IQR)	4 (3–17)	4 (2–14)	0.685^#^
Year after RFT, median (IQR)	3 (1–15)	3 (1–13)	0.803^#^
History of Reaction, N (%)	19 (63)	27 (49)	0.208
Type 1 reaction	4 (13)	10 (18)	
Type 2 reaction	8 (27)	10 (18)	
Neuritis	7 (23)	7 (13)	
Current Reaction, N (%)	15 (50)	14 (26)	0.023*
Type 1 reaction	2 (7)	1(2)	
Type 2 reaction	7 (23)	10 (18)	
Neuritis	6 (13)	3 (5)	
Relapse, N (%)	1 (3)	3 (5)	1.000^#^
Nerve thickening, N (%)	21 (70)	40 (73)	0.790
Nerve tenderness, N (%)	12 (40)	10 (18)	0.028*
WHO disability grade, N (%)			0.312
0	7 (23)	13 (24)	
1	9 (30)	9 (16)	
2	14 (47)	33 (60)	
EHF score, mean ± SD	3.4 ± 2.8	3.7 ± 3.3	0.667
Nerve Function Impairment, N (%)	23 (77)	43 (78)	0.873

^#^, Fischer’s exact test or Mann-Whitney test

*, *p* < 0.05; RFT, release from treatment; SD, standard deviation; IQR, interquartile range.

**Fig 2:**
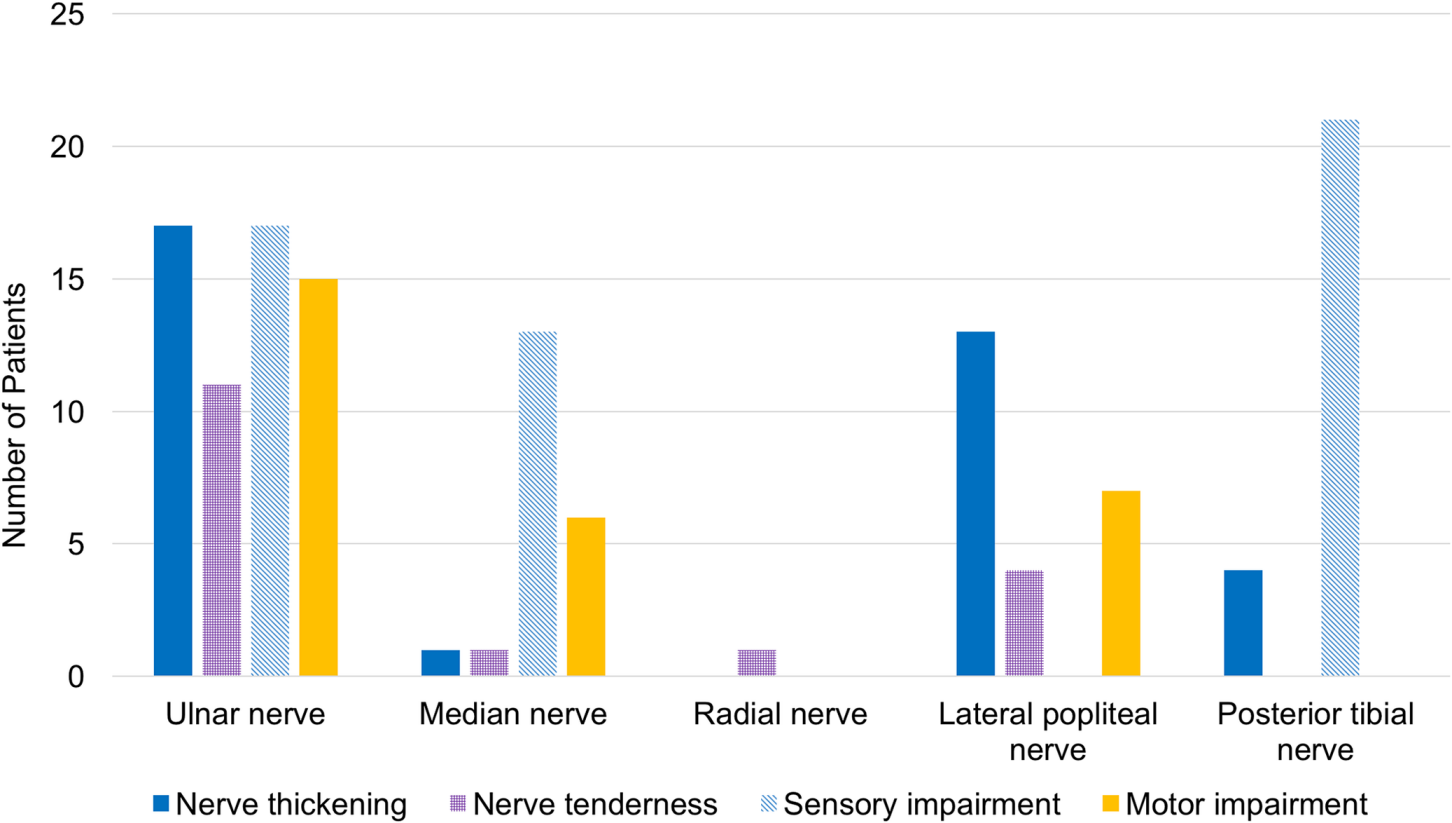
Peripheral nerve involved in RFT patients with neuropathic pain (total 30 patients).

In RFT patients with NP, 21 (70%) had nerve thickening and 12 (40%) had nerve tenderness on physical examination. The ulnar nerve was the most predominantly affected nerve in our study population (Fig 2). The prevalence of NFI at diagnosis and the degree of disability were similar regardless of the presence of current NP.

**Fig 3:**
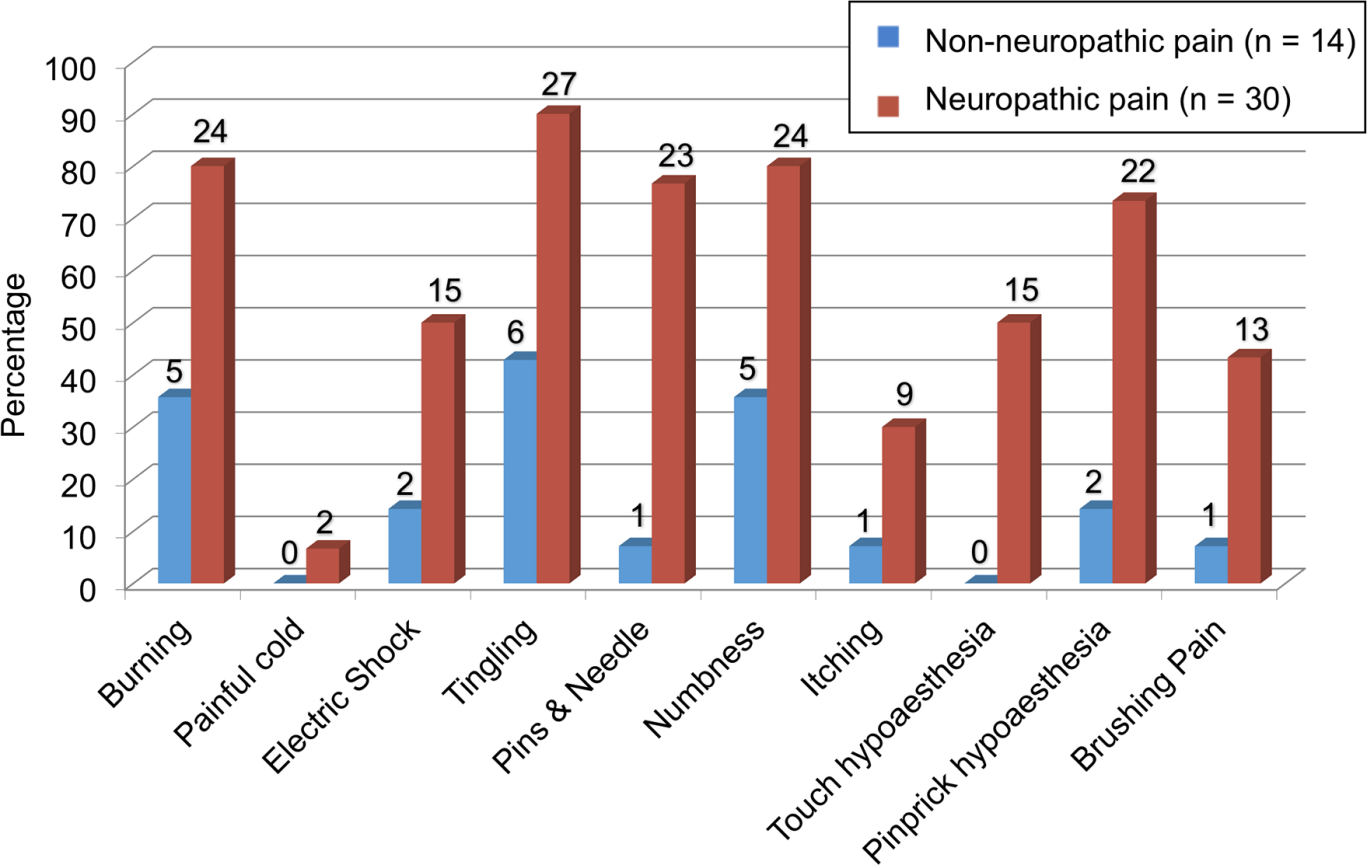
DN4 questionnaire results in RFT patients with and neuropathic and non-neuropathic pain.

Of the symptoms tested by the DN4 questionnaire, the most common was tingling (90%, 27/30), followed by burning (80%, 24/30) and numbness (80%, 24/30) (Fig 3). On physical examination, 73% (22/30) had pinprick hypoesthesia and 50% (15/30) had touch hypoesthesia in the area where they complained of pain. Allodynia was demonstrated in 43% (13/30) of the patients with NP.

### Pain severity

**Fig 4:**
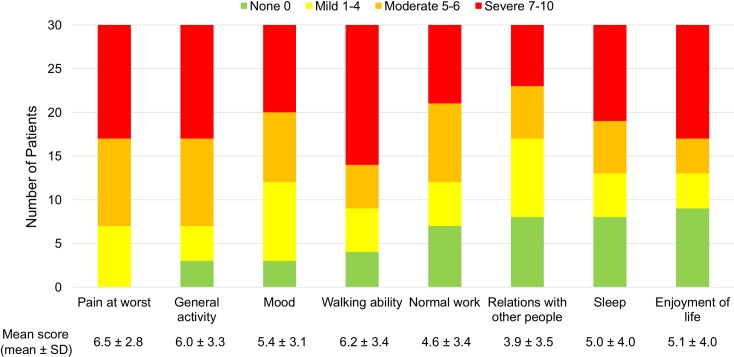
Pain severity and daily life interference in patients with neuropathic pain.

Among RFT patients with NP at the time of interview, 50% (15/30) suffered from moderate pain and 37% (11/30) with severe pain. The mean worst pain score was higher in NP compared to non-NP RFT patients (6.5 ± 2.8 vs. 4.4 ± 3.1). Concerning pain interference, those with NP had a moderate degree of daily life disturbance with a mean interference score of 5.2 ± 2.7, comparing to that of non-NP group (2.1 ± 2.3, *p* = 0.003). More than half had a moderate to severe degree of interference in general activity, mood, walking ability, normal work, sleep and enjoyment of life (Fig 4). However, only 63% (19/30) were receiving pain management, including 8 patients with acetaminophen, 7 patients with amitriptyline, 3 with gabapentin and 1 with non-steroidal anti-inflammatory drug. The overall response to medications was only moderate, with an average 57% pain reduction with amitriptyline and 67% with gabapentin. Therefore, while NP caused moderate to severe physical and psychological interferences, medications only helped to a moderate degree (50–60%) in reduction of pain.

### Activity limitation

**Fig 5:**
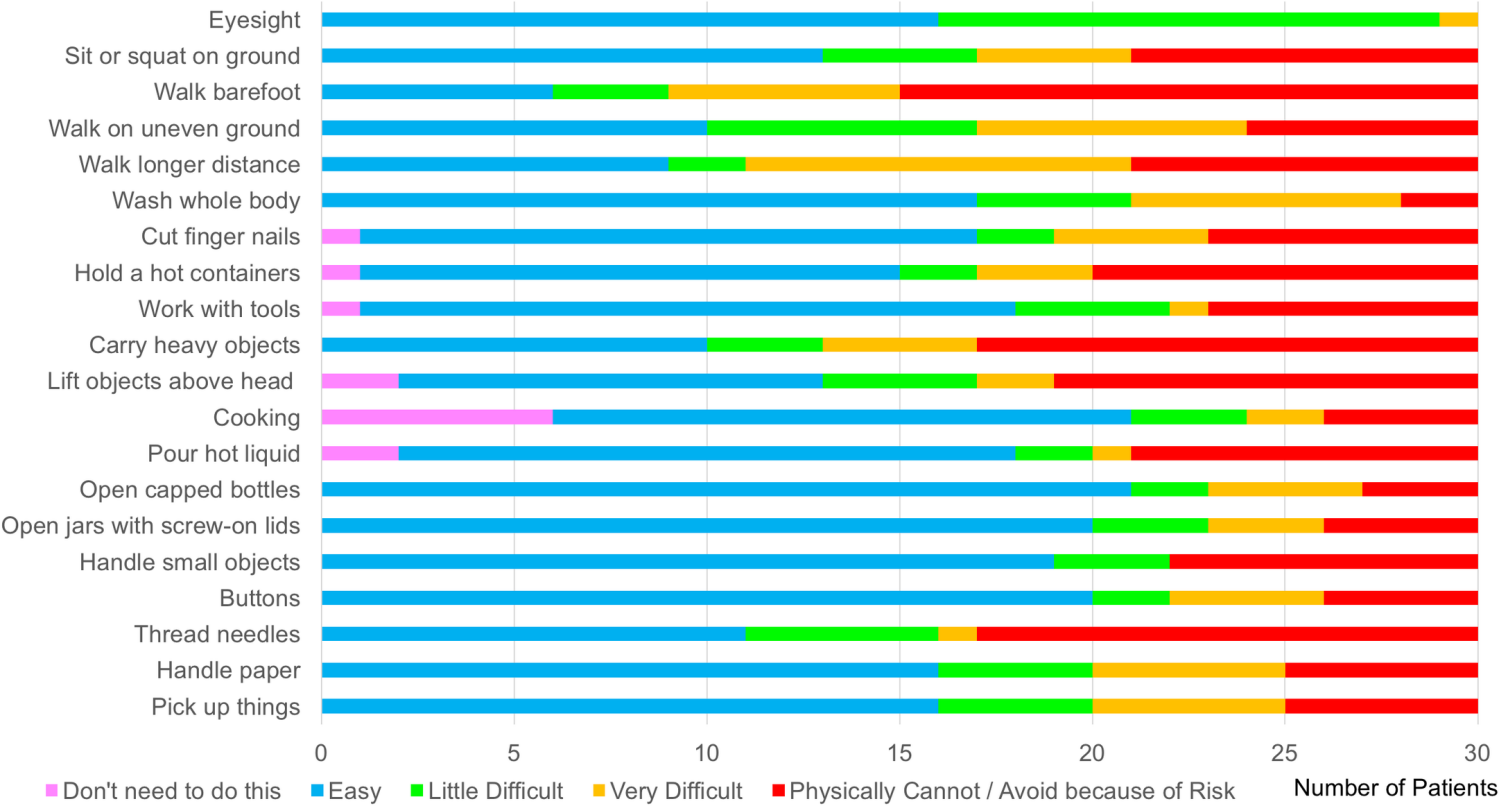
SALSA score of individual items of the patients with neuropathic pain.

In RFT patients with NP, 83% (25/30) had some level of activity restriction in their daily life and nearly half (47%, 14/30) had moderate to extreme limitation (Table 2). In comparison, this degree of limitation only occurred in 18% (10/55) of RFT patients without NP (*p* = 0.005). The most common problem in RFT patients with NP was walking barefoot, as 80% found it difficult or they just physically could not do it (Fig 5). SALSA score correlated poorly to pain interference score (F(1, 28) = 0.70, r-squared 0.025, p = 0.408) within NP group. Nevertheless, the mean SALSA score of those with NP was significantly higher than patients without NP (41.4 ± 17.0 vs. 32.6 ± 14.3, *p* = 0.013). However, the mean EHF score and G2D rates were similar in both groups. This result highlights that even with similar degree of disabilities, those with NP experienced more activity restriction.

**Table 2:** Degree of activity limitation for RFT patients with and without neuropathic pain.

Patient Group	Degree of Activity Limitation (SALSA score)				
	No (15–24)	Mild (25–39)	Moderate (40–49)	Severe (50–59)	Extreme (60–80)
Neuropathic Pain (n = 30)	5 (17%)	11 (33%)	4 (13%)	4 (13%)	6 (18%)
Without neuropathic pain (n = 55)	18 (32%)	27 (49%)	3 (5%)	1 (3%)	6 (11%)

### Psychological morbidity

With the GHQ-12, the mean score for all RFT patients was 10.2 ± 7.7 when using the Likert-type scale. A cut-off point of 11 was used to identify patients with psychological distress. 30 (35%) RFT patients were found to have some degree of psychological distress, of which 13 (13/30, 43%) also had NP. Only 20% (6/30) of them had current reaction, but 73% (22/30) had G2D and 50% (15/30) had moderate to extreme activity limitation. 43% (13/30) of RFT patients with NP demonstrated GHQ-12 scores that indicated psychological distress compared to 31% (17/55) of RFT cases without NP; however, this difference was not significant (*p* = 0.252).

### Multivariate analysis

Within this cohort, NP was not associated with any presentation variables taken at leprosy diagnosis, including: gender, age, classification, presence of skin lesions or nerve involvement (Table 1). At the time of ongoing NP, only symptoms of nerve tenderness and current reaction demonstrated association with *p* < 0.05. A stepwise forward multivariate logistic regression was then performed, which indicated that NP is associated with nerve tenderness (OR 3.40, 95%CI 1.19–9.76, *p* = 0.023) and current reactions (OR 3.28. 95%CI 1.23–8.77, *p* = 0.018).

## Discussion

Although leprosy is known for neuropathy leading to sensory loss, pain is a common complaint in patients with leprosy and often clinically ignored in standardized assessments [33]. Within our study cohort of Nepalese RFT patients, about 52% (44/85) still experience pain and 35% (30/85) have NP in the years after completion of MDT, a finding which is compatible with previous studies in other endemic settings [21, 22]. Studies in other endemic populations have indicated that NP prevalence was lower (11.3–26.2%) when RFT patients were screened beyond those attending hospital services for ongoing complications [7, 11, 12, 23]. Nevertheless, NP was detected in a significant proportion of RFT patients seeking clinical care and, therefore, represents an issue of noteworthy concern.

Differentiation between nociceptive pain and NP is important as therapeutic options may vary. However, a patient and field-friendly standardized screening tool could be helpful for leprosy clinicians. By using the DN4 questionnaire, we found that tingling (in Nepalese “jham-jham”) was the most common term used by RFT patients (90%) to describe NP, followed closely by burning (80%) and numbness (80%). However, this result could perhaps vary depending on cultural and language inferences across different endemic populations. In addition, the DN4 has been shown to sometimes underestimate NP prevalence with a predictive value of 86% or even lower in some settings [13, 15]. Therefore, a better tool or protocol is needed to assist in the standardized clinical diagnosis of NP internationally.

Pain measurement is crucial to understanding the severity and effects of NP. Within our RFT patient group, the BPI-short did provide a quantitative method of pain measurement, including response to medications. Our study demonstrated that NP severity and related interference in daily life is much more severe than with other types of pain reported. Moreover, our RFT patients reported only moderate response to tricyclic antidepressants and anticonvulsants, which are regarded as the first-line treatments for NP. A recent study by Raicher et al showed that the pain characteristics of NP due to leprosy are similar to NP cause by other etiologies, and their management should not be different [34]. However, there is still a lack of evidence for medical treatment of NP in leprosy patients; and pain scoring is essential to best identify dose and titration schedules according to individual patient needs and response to therapy.

In our study, we used SALSA scores to measure the degree of activity limitation for correlation with NP. Compared to previous studies, a lower proportion of RFT patients with NP had moderate to extreme limitation in our study (46.7% vs. 66.7%) [20]. Regardless, they reported more physical restrictions than RFT patients without NP; and there was no difference in disability grade proportions between groups. This highlights that NP itself can cause significant decrease in functional capacity regardless of the degree of disability. We do not know whether this effect is reversible, and more studies are needed to show if there is a restoration of function in patients with NP after treatment.

Psychological morbidity is known to be associated with both leprosy and NP [18, 32, 33]. Reporting of pain can also be influenced by psychological and sociocultural factors such as stigma. In our study, there was a trend of RFT patients with NP to more often exhibit signs of psychological distress than RFT patients without NP. A more comprehensive evaluation would be required to confirm possible clinical levels of psychiatric illness (i.e., depression). Treatment with antidepressant or other psychotropic medications should be considered in such situations as the subjective perception of pain is usually aggravated by depression [35, 36].

The ability to predict which leprosy patients may suffer NP after RFT may be limited, as none of the other variables from initial leprosy diagnosis demonstrated association. Mechanisms of NP involve a complicated neural pathway from peripheral afferent nerves to the central nervous system. The potential for *M*. *leprae* and its antigenic residues to persist along peripheral nerve pathways creates neuropathic vulnerability for all patients, although risk for occurrence would be anticipated to be similar to reaction development risks (i.e., increased risk for those with T2R, which usually associated with higher *M*. *leprae* loads) [10]. Therefore, prevention of NP also likely best coincides with early detection and treatment of leprosy.

Finally, our analysis revealed that nerve tenderness and ongoing reactions are significant risk factors for the development of NP. The study by Lasry-Levy et al. in India also demonstrated that nerve tenderness is a risk factor for NP [9]. Considering the mechanisms of leprosy neuropathy, it is puzzling that some NP studies have excluded patients with reactions, considering them a confounding rather than contributing factor for NP [22]. Another study indicated that ongoing or new onset neuritis is mostly caused by reactions or relapse, and that it could present with pain or NFI [16]. Early recognition and management of neuritis are crucial to avoid further nerve damage and complications, including NP.

There were limitations within this study. First of all, this was an opportunistic cross-sectional study of RFT patients attending leprosy referral services for ongoing issues; therefore, it likely does not represent NP incidence for all RFT patients. People without any complication after RFT would not attend to the hospital and thus, our study population represents leprosy-affected people that developed more clinical issues. However, it is important to emphasize the importance of NP while taking care of the patients who completed MDT in those settings. Prospective studies with larger sample sizes could improve statistical accuracy for broader estimations.

On the other hand, there was risk for some bias within the study. First to mention is the possible underestimation of NP prevalence. Although DN4 has a relatively good predictive accuracy for NP, it might fail to detect NP in 10–20% of patients compared to clinical diagnosis according to the IASP guideline [15]. However, these screening tools are standard in this field, and there are currently no other better tools available. Secondly, patient's self-reported answers for years past might have recall and reporting bias. Contextual circumstances (natural disasters, war, etc.), as well as cultural perspectives regarding expression of pain and psychological problems may shadow responses and limit broader generalizations among diverse settings. For instance, at the time of interview, many Nepalese patients were still dealing with traumatic outcomes from the 2015 earthquakes and border blockades such as displacement, not yet being able to rebuild their homes, and loss of family members or livelihood–contextual issues that may have cast leprosy and NP with a lesser comparative burden for psychological impact. Especially notable during the GHQ-12 questionnaire, we usually received a conservative response regarding emotional expression, which could have resulted in an underestimate of psychological impact.

Nevertheless, our results clearly emphasize the importance of pain and NP in regard to leprosy clinical care. Foremost, a significant proportion of RFT patients suffer from NP years after technical cure; therefore, NP should be more often considered during formal assessment. Appropriate patient counseling to set realistic expectations prior to RFT could also better prepare patients in advance to report NP and reaction issues early for management, thereby potentially lowering risk for disability development.

### Conclusions

Our findings demonstrate that even years after RFT, 76% of patients reported history of pain and 52% report ongoing pain, of which roughly two-thirds were found to have NP. Patients with NP suffer from more severe pain and more physical restrictions in their daily life. The response to medical therapy is only moderate. There is also a significant proportion of NP sufferers exhibiting psychological distress, which may require interventions other than pain control.

In conclusion, there is currently no method to predict or prevent NP in leprosy patients; and mechanisms of NP development are poorly understood. There is a dearth of evidence for how to effectively treat NP. Randomized controlled trials are needed to provide reliable evidence for NP treatment in leprosy patients and for relevant impact on activity limitations and psychological well-being. To provide comprehensive care, leprosy clinicians need to have contextually appropriate and reliable screening tools for detecting NP and measuring its impact. The DN4 is currently the best single tool for diagnosis of NP, but the tool has limitations and risks of underestimation. Pain measurement indicators such as BPI-short are mandatory to determine pain severity in reference for therapeutic decisions and response management. NP significantly impacts activity limitation and psychological morbidity, for which SALSA and GHQ-12 scores could be used.

## Supporting information

S1 Checklist STROBE checklist. (DOC)
